# Multifactorial methods integrating haplotype and epistasis effects for genomic estimation and prediction of quantitative traits

**DOI:** 10.3389/fgene.2022.922369

**Published:** 2022-10-14

**Authors:** Yang Da, Zuoxiang Liang, Dzianis Prakapenka

**Affiliations:** Department of Animal Science, University of Minnesota, Saint Paul, MN, United States

**Keywords:** multifactorial model, epistasis, haplotype, SNP, GBLUP, GREML

## Abstract

The rapid growth in genomic selection data provides unprecedented opportunities to discover and utilize complex genetic effects for improving phenotypes, but the methodology is lacking. Epistasis effects are interaction effects, and haplotype effects may contain local high-order epistasis effects. Multifactorial methods with SNP, haplotype, and epistasis effects up to the third-order are developed to investigate the contributions of global low-order and local high-order epistasis effects to the phenotypic variance and the accuracy of genomic prediction of quantitative traits. These methods include genomic best linear unbiased prediction (GBLUP) with associated reliability for individuals with and without phenotypic observations, including a computationally efficient GBLUP method for large validation populations, and genomic restricted maximum estimation (GREML) of the variance and associated heritability using a combination of EM-REML and AI-REML iterative algorithms. These methods were developed for two models, Model-I with 10 effect types and Model-II with 13 effect types, including intra- and inter-chromosome pairwise epistasis effects that replace the pairwise epistasis effects of Model-I. GREML heritability estimate and GBLUP effect estimate for each effect of an effect type are derived, except for third-order epistasis effects. The multifactorial models evaluate each effect type based on the phenotypic values adjusted for the remaining effect types and can use more effect types than separate models of SNP, haplotype, and epistasis effects, providing a methodology capability to evaluate the contributions of complex genetic effects to the phenotypic variance and prediction accuracy and to discover and utilize complex genetic effects for improving the phenotypes of quantitative traits.

## Introduction

Genomic estimation of variance components and associated heritabilities and genomic prediction for quantitative traits using single nucleotide polymorphism (SNP) markers and mixed models have become a widely used approach for genetic improvement in livestock and crop species. The rapid growth in genomic selection data provides unprecedented opportunities to discover and utilize complex genetic mechanisms, but methodology and computing tools are lacking for investigating complex genetic mechanisms using the approach of genomic estimation and prediction. The integration of global low-order epistasis effects and local high-order epistasis effects contained in haplotypes for genomic estimation and prediction is a step forward for the discovery and application of complex genetic mechanisms to improve the phenotypes of quantitative traits.

The theory of genetic partition of two-locus genotypic values defines four types of epistasis values: additive × additive (A×A), additive × dominance (A×D), dominance × additive (D×A), and dominance × dominance (D×D) epistasis values (Cockerham, 1954; Kempthorne, 1954). The Cockerham method defines each epistasis coefficient as the product of the coefficients of the two interacting effects that each can be an additive or dominance effect (Cockerham, 1954). This definition of epistasis coefficient is the basis for defining epistasis model matrices in terms of the model matrices of additive and dominance effects. Cockerham also defines a pedigree epistasis relationship as the product between the pedigree additive and dominance relationships (Cockerham, 1954), and this definition is the theoretical basis for Henderson’s approach to express epistasis relationship matrices as the Hadamard products of the additive and dominance relationship matrices (Henderson, 1985). Henderson’s Hadamard products for epistasis relationship matrices were suggested for genomic prediction using epistasis effects by replacing the pedigree additive and dominance relationship matrices with the genomic additive and dominance relationship matrices calculated from SNP markers (Su et al., 2012; Muñoz et al., 2014; Vitezica et al., 2017). This genomic version of Henderson’s Hadamard products avoids the use of large epistasis model matrices that can be difficult or impossible to compute but contains intra-locus epistasis effects that are not present in the epistasis model (Martini et al., 2020). For this reason, the genomic version of Henderson’s Hadamard products could be described as approximate genomic epistasis relationship matrices (AGERM). Formulations have been developed to obtain the exact genomic epistasis relationship matrices (EGERM) that remove the intra-locus epistasis effects in AGERM by modifying Henderson’s Hadamard products without creating the epistasis model matrices (Jiang and Reif, 2015; Martini et al., 2016; Jiang and Reif, 2020; Martini et al., 2020). The difference between AGERM and EGERM tends to diminish as the number of SNPs increases (Jiang and Reif, 2020). Henderson’s Hadamard products and hence AGERM are applicable to any order of epistasis effects, and EGERM also has a general formula for any order of epistasis effects (Jiang and Reif, 2020). However, limited tests showed that fourth-order global epistasis contributed virtually nothing to the phenotypic variance but generated considerable computing difficulty (Liang et al., 2021), raising questions about the value of global epistasis effects beyond the third-order. Methods of genomic estimation and prediction of global epistasis effects up to the third-order should have wide-range applications, given that the number of reported epistasis effects lags far behind the number of single-point effects (Carlborg and Haley, 2004; Phillips, 2008; Ritchie and Van Steen, 2018) even though epistasis effects are important genetic effects (Cordell, 2002; Segre et al., 2005; Mackay, 2014). In contrast to the computing difficulty and uncertain impact of global high-order epistasis effects beyond the third-order, local high-order epistasis effects in haplotypes with potentially many SNPs were responsible for the increased accuracy of predicting phenotypic values of certain traits (Liang et al., 2020; Bian et al., 2021). The integration of haplotype and epistasis effects provides an approach to investigate the contributions of global low-order epistasis effects and local high-order epistasis effects to the phenotypic variance and the accuracy of genomic prediction under the same model.

An epistasis GWAS in Holstein cattle showed that intra- and inter-chromosome epistasis effects affected different traits differently, for example, the daughter pregnancy rate was mostly affected by inter-chromosome epistasis effects, whereas milk production traits were mostly affected by intra-chromosome epistasis effects (Prakapenka et al., 2021), and genomic heritability estimates of intra- and inter-chromosome heritabilities for the daughter pregnancy rate using methods in this article showed that inter-chromosome A×A heritability was much higher than the intra-chromosome A×A heritability (Liang et al., 2022). Therefore, dividing pairwise epistasis effects into intra- and inter-chromosome epistasis effects allows the investigation of the contributions of intra- and inter-chromosome pairwise epistasis effects to the phenotypic variance and prediction accuracy.

The purpose of the multifactorial model in this article is to integrate haplotype effects and epistasis effects up to the third-order for genomic estimation of variance components and associated heritabilities, as well as genomic prediction of genetic and phenotypic values of quantitative traits, to provide a general and flexible methodology framework for genomic prediction and estimation using complex genetic mechanisms and to provide methodology details of the EPIHAP computer package that implements the integration of haplotype and epistasis effects (Liang et al., 2021, 2022). The methodology in this article will facilitate the discovery and utilization of global low-order and local high-order epistasis effects relevant to the phenotypic variances and prediction accuracies of quantitative traits, and obtain new knowledge of complex genetic mechanisms underlying quantitative traits.

## Materials and methods

### Quantitative genetics model with single nucleotide polymorphism, haplotype, and epistasis effects and values

The mixed model with single-SNP additive and dominance effects, haplotype additive effects, and pairwise SNP epistasis effects in this article is based on the quantitative genetics (QG) model resulting from the genetic partition of single-SNP genotypic values (Da et al., 2014; Wang and Da, 2014), haplotype genotypic values (Da, 2015), and pairwise genotypic values (Cockerham, 1954). An advantage of this QG model is the readily available quantitative genetics interpretations of SNP additive and dominance effects, values, and variances; haplotype additive effects, values, and variances; epistasis effects, values, and variances; and the corresponding SNP, haplotype, and epistasis heritability estimates. Two QG models are developed: Model-I with 10 effect types, including SNP additive and dominance effects, haplotype additive effects, and epistasis effects up to the third-order; and Model-II with 13 effect types resulting from replacing the pairwise epistasis effects of Model-I with intra- and inter-chromosome epistasis effects. Detailed descriptions of the effects, values, model matrices, the coding of the model matrices, as well as the precise definition of each term in the two QG models, are provided in Supplementary Text S1 and Supplementary Table S1. With these precise definitions of genetic effects, values, and model matrices in the QG models, a concise multifactorial QG model covering both Model-I and Model-II can be established, that is

$$g=\mu I+\sum_{i=1}^{f}W_{i}\tau_{io}=\mu I+\sum_{i=1}^{f}u_{i}$$ (1)

$$u_{i}=W_{i}\tau_{io}$$ (2)

where $\tau_{io}=$ genetic effects of the $i^{th}$ effect type from the original QG model based on genetic partition, $W_{i}=$ model matrix of $\tau_{io}$ , $u_{i}=$ genetic values of the $i^{th}$ effect type from the original QG model, and f = number of effect types. For Model-I, subscripts i=1,…,10 represent SNP additive (A), SNP dominance (D), haplotype additive, A×A, A×D, D×D, A×A×A, A×A×D, A×D×D, and D×D×D effects sequentially. For Model-II, subscripts i=1,…,13 represent SNP additive, SNP dominance, haplotype additive, intra-chromosome A×A, intra-chromosome A×D, intra-chromosome D×D, inter-chromosome A×A, inter-chromosome A×D, inter-chromosome D×D, A×A×A, A×A×D, A×D×D, and D×D×D effects sequentially. The variance–covariance matrix of the genetic values of Eqs 1 and 2 is

$$G=var\left(\sum_{i=1}^{f}W_{i}\tau_{io}\right)=\sum_{i=1}^{f}Var\left(u_{i}\right)=\sum_{i=1}^{f}G_{i}=\sum_{i=1}^{f}\sigma_{io}^{2}W_{i}W_{i}^{\prime }$$ (3)

$$Var\left(\tau_{io}\right)=\sigma_{io}^{2}I$$ (4)

$$G_{i}=Var\left(u_{i}\right)=W_{i}Var\left(\tau_{io}\right)W_{i}^{\prime }=\sigma_{io}^{2}W_{i}W_{i}^{\prime }$$ (5)

where $\sigma_{io}^{2}=Var\left(\tau_{ijo}\right)$ genetic variance of the $i^{th}$ effect type under the original QG model is common to all individuals (all j values). It is of note that $W_{i}{W_{i}}^{\prime }$ is not a genomic relationship matrix but is the primary information for calculating each genomic relationship matrix. The structure of the **G** matrix of Eqn. 3 assumes independence between the genetic values of different effect types. However, the GBLUP values of different effect types using the **G** matrix of Eqn. 3 could be correlated. Under the Hardy–Weinberg equilibrium (HWE) and LE assumptions, additive, dominance, and epistasis effects are independent of each other (Cockerham, 1954; Kempthorne, 1954). For genome-wide SNPs, the LE assumption generally does not hold for closely linked loci, and nonzero Hardy–Weinberg disequilibrium (HWD) may exist numerically. These and other unknown factors in real data may result in the existence of correlations between different effect types. Haplotype additive values are correlated with SNP additive effects because a haplotype additive value is the sum of all SNP additive values and an epistasis value within the haplotype block plus a potential haplotype loss (Da et al., 2016). In two recent haplotype studies for genomic prediction, the integration of SNP and haplotype effects increased the prediction accuracy for four of the seven traits in the human study (Liang et al., 2020) and for three of the eight traits in the swine study (Bian et al., 2021), showing that SNP and haplotype additive values compensated each other for prediction accuracy and that the correlation between SNP and haplotype additive values were incomplete for those traits. The correlation between haplotype and epistasis values can be complex. The correlation should be nonexistent if the A×A values are inter-chromosome A×A values or intra-chromosome A×A values involving distal SNPs not covered by the haplotypes, but the correlation could be strong if the A×A values are intra-chromosome A×A values involving proximal SNPs covered by the haplotypes.

### The reparametrized and equivalent quantitative genetics model for genomic estimation and prediction

Genomic relationship matrices are used for genomic estimation and prediction, and the use of genomic relationship matrices results in a reparametrized and equivalent model of the original QG model for genetic values, to be referred to as the RE-QG model, where “reparametrized” refers to the reparameterization of the genetic effects, model matrix, and genetic variance of each effect type; and “equivalent” refers to the requirement of the same first and second moments for the original QG model (Eqs 1–5) and the RE-QG model. This RE-QG model of genetic values can be expressed as

$$g=\mu I+\sum_{i=1}^{f}T_{i}\tau_{i}=\mu I+\sum_{i=1}^{f}u_{i}$$ (6)

$$G=var\left(\sum_{i=1}^{f}u_{i}\right)=\sum_{i=1}^{f}G_{i}=\sum_{i=1}^{f}\sigma_{i}^{2}T_{i}T_{i}^{\prime }=\sum_{i=1}^{f}\sigma_{i}^{2}S_{i}=\sum_{i=1}^{f}\sigma_{io}^{2}W_{i}W_{i}^{\prime }$$ (7)

where

$$\tau_{i}=\sqrt{k_{i}}\tau_{io}=genetic\;effects\;of\;the\;i^{th}\;effect\;type$$ (8)

$$T_{i}=W_{i}/\sqrt{k_{i}}=model\;matrix\;of\;\tau_{i}$$ (9)

$$\sigma_{i}^{2}=Var\left(\tau_{ij}\right)=tr\;\left(G_{i}\right)/n=\sum_{j=1}^{n}G_{i}^{jj}/n=k_{i}\sigma_{io}^{2}$$ (10)

= variance of the genetic effects of the $i^{th}$ effect type common to all individuals= average variance of all individuals for the genetic values of the $i^{th}$ effect type

$$u_{i}=T_{i}\tau_{i}=W_{i}\tau_{io}=genetic\;values\;of\;the\;i^{th}\;effect\;type$$ (11)

$$G_{i}=Var\left(u_{i}\right)=\sigma_{i}^{2}T_{i}T_{i}^{\prime }=\sigma_{i}^{2}S_{i}=\sigma_{io}^{2}W_{i}W_{i}^{\prime }$$ (12)

= variance–covariance matrix of the genetic values of the $i^{th}$ effect type

$$S_{i}=T_{i}T_{i}^{\prime }=W_{i}W_{i}^{\prime }/k_{i}=genomic\;relationship\;matrix\;of\;the\;i^{th}\;effect\;type$$ (13)

$$k_{i}=tr\left(W_{i}W_{i}^{\prime }\right)/n=average\;of\;the\;diagonal\;elements\;ofW_{i}W_{i}^{\prime }.$$ (14)

Equations 8–10 are the reparametrization of the genetic effects, model matrices, and genetic variances of the original QG model, whereas Eqs 11 and 12 show the genetic values and the variance–covariance matrix of the genetic values are the same under the RE-QG and QG models. In Eq.10, $G_{i}^{jj}$ = the genetic variance of the $j^{th}$ individual for the $i^{th}$ effect type = the $j^{th}$ diagonal element of the $G_{i}$ matrix defined by Eq. 12. The $k_{i}$ formula of Eq. 14 as the average of the diagonal elements of $W_{i}W_{i}^{\prime }$ was originally proposed for genomic additive relationships (Hayes and Goddard, 2010) and was used for genomic dominance relationships (Da et al., 2014; Wang and Da, 2014), haplotype additive genomic relationships (Da, 2015), and pairwise epistasis genomic relationships (Vitezica et al., 2017). The need for this RE-QG model is due to the use of the genomic relationship matrices (e.g., Eq. 13) because the QG model does not contain genomic relationship matrices (Eq. 3). Detailed notations of the QG model of Eqs 1–5 in reference to the RE-QG model described by Eqs 6–14 are summarized in Supplementary Table S1.

The formula of the genomic relationship matrix ( $S_{i}$ of Eq. 13) is based on the model matrix of each effect type and can be difficult or impossible to compute if epistasis model matrices are used. This computing difficulty of epistasis model matrices is removed by calculating $S_{i}$ based on the model matrices of SNP additive and dominance effects without creating the epistasis model matrices using either AGERM or EGERM. AGERM refers to the genomic version of Henderson’s Hadamard products between pedigree additive and dominance relationship matrices (Henderson, 1985), with the pedigree additive and dominance relationship matrices replaced by the genomic additive and dominance relationship matrices (Su et al., 2012; Muñoz et al., 2014; Vitezica et al., 2017). AGERM contains intra-locus epistasis that should not exist (Martini et al., 2020), and EGERM removes intra-locus epistasis from AGERM based on products between genomic additive and dominance relationship matrices (Jiang and Reif, 2020; Martini et al., 2020).

The QG and RE-QG models have the same prediction accuracy due to the equivalence between these two models. The genetic values ( $u_{i}$ , Eqs 2, 11) and the variance–covariance matrix of the genetic values ( $G_{i}$ , Equations 5 and 12) under the QG and RE-QG models are identical, although $u_{i}$ and $G_{i}$ have different expressions under the QG and RE-QG models. Consequently, the QG model without using genomic relationship matrices and the RE-QG model using genomic relationship matrices have identical accuracy of genomic prediction. The choice of the $k_{i}$ formula for defining the genomic relationship matrix does not affect the accuracy of genomic prediction but affects the interpretation and application of the genetic variance and genomic relationships for each effect type. Since the interpretation of each genetic variance is a focus, whereas the interpretation of the genomic relationships is not a focus in this study, the interpretation of the genetic variance and associated heritability is the consideration in choosing the $k_{i}$ formula of Eq.14.

The RE-QG model using genomic relationships (Equations 6–14) has two major advantages over the QG model without using genomic relationship matrices (Equations 1–5), although the two models have the same prediction accuracy. First, the use of genomic relationships, originally proposed for genomic additive relationships (VanRaden, 2008), provides a genomic version of the traditional theory and methods of best linear unbiased prediction (BLUP) that uses pedigree relationships, and this genomic version can utilize a wealth of BLUP-based theory, methods, and computing strategies. Second, the genetic variance of the genetic effects of each effect type under the RE-QG model can be used for estimating genomic heritability, whereas the genetic variance of the genetic effects under the QG model cannot be used for estimating genomic heritability. With the $k_{i}$ value defined by Eq. 14, the variance of the genetic effects of the $i^{th}$ effect type, $\sigma_{i}^{2}=k_{i}\sigma_{io}^{2}$ (Eq.10), has the unique interpretation as the average variance of the genotypic values of all individuals and is a common variance to all individuals. Moreover, $\sigma_{i}^{2}=k_{i}\sigma_{io}^{2}$ is unaffected by the number of levels for each effect type, unless the number of levels such as the number of SNPs is too small to provide sufficient coverage of the genome (Da et al., 2014; Tan et al., 2017; Liang et al., 2020). In contrast, the QG model does not have a method to estimate genetic variance components for calculating genomic heritabilities because $\sigma_{io}^{2}$ is an inverse function of the number of effect levels. As the number of effect levels such as the number of SNPs increases or decreases, the value of each element in $W_{i}W_{i}^{\prime }$ changes in the same direction and the $\sigma_{io}^{2}$ estimate changes in the opposite direction, that is, as the number of effect levels increases or decreases, $\sigma_{io}^{2}$ decreases or increases. Consequently, the $\sigma_{io}^{2}$ estimate does not have a unique interpretation and cannot be used for estimating genomic heritability (Da et al., 2014). Moreover, the variance of the genetic value of an individual $\left(\sigma_{io}^{2}{\left(W_{i}W_{i}^{\prime }\right)}^{jj}\right)$ cannot be used for calculating genomic heritability because of the individual specificity of the ${\left(W_{i}W_{i}^{\prime }\right)}^{jj}$ values, as shown as follows.

The exact relationship between the genetic variance for the $i^{th}$ effect type of the $j^{th}$ individual under the RE-QG model and the QG model can be described based on the $G_{i}$ matrix defined by Eq. 12:

$$G_{i}^{jj}=Var\left(u_{ij}\right)=\sigma_{i}^{2}{\left(S_{i}\right)}^{jj}=\sigma_{io}^{2}{\left(W_{i}W_{i}^{\prime }\right)}^{jj}$$ (15)

where $G_{i}^{jj}=$ the $j^{th}$ diagonal element of the $G_{i}$ matrix defined by Eq.12 = the genetic variance of the $j^{th}$ individual for the genotypic value of the $i^{th}$ effect type, and $u_{ij}$ = the $j^{th}$ element of $u_{i}$ defined by Eq.11. Equation 15 shows that different individuals do not have a common variance of the genetic values $\left(G_{i}^{jj}\right)$ unless all diagonal elements of $S_{i}$ or $W_{i}W_{i}^{\prime }$ are identical, which could not happen with genome-wide SNP data in the absence of identical twins because genome-wide SNPs have a high degree of individual specificity. Consequently, $G_{i}^{jj}$ is not a common variance to all individuals and cannot be used for calculating the genomic heritability of the $i^{th}$ effect type. In contrast, $\sigma_{i}^{2}$ of Eq.10 under the RE-QG model as the average variance of the genotypic values of all individuals is common to all individuals and can be used for calculating the heritability of each effect type. For the example of Model-I, the exact genetic interpretation of $G_{i}^{jj}$ is $G_{i}^{jj}=\sigma_{aj}^{2}=$ the variance of the genomic additive (breeding) value of the $j^{th}$ individual for i=1 , $G_{i}^{jj}=\sigma_{dj}^{2}=$ the variance of the genomic dominance value of the $j^{th}$ individual for i=2 , $G_{i}^{jj}=\sigma_{ahj}^{2}=$ the variance of the genomic haplotype additive value of the $j^{th}$ individual for i=3 , $G_{i}^{jj}=\sigma_{aaj}^{2}=$ the variance of the A×A value of the $j^{th}$ individual for i=4 , $G_{i}^{jj}=\sigma_{adj}^{2}=$ the variance of the A×D value of the $j^{th}$ individual for i=5 , $G_{i}^{jj}=\sigma_{ddj}^{2}=$ the variance of the D×D value of the $j^{th}$ individual for i=6 , $G_{i}^{jj}=\sigma_{aaaj}^{2}=$ the variance of the A×A×A value of the $j^{th}$ individual for i=7 , $G_{i}^{jj}=\sigma_{aadj}^{2}=$ the variance of the A×A×D value of the $j^{th}$ individual for i=8 , $G_{i}^{jj}=\sigma_{addj}^{2}=$ the variance of the A×D×D value of the $j^{th}$ individual for i=9 , and $G_{i}^{jj}=\sigma_{dddj}^{2}=$ the variance of the D×D×D value of the $j^{th}$ individual for i=10 . These genetic interpretations, along with those for intra- and inter-chromosome pairwise epistasis effects of Model-II under the QG and RE-QG models, are summarized in Supplementary Table S1.

## Results and discussion

### The multifactorial model of phenotypic values

Based on the RE-QG model of Eqs 6–14, the multifactorial model for phenotypic values is

$$\begin{array}{l} y=Xb+Zg+e=Xb+Z\sum_{i=1}^{f}T_{i}\tau_{i}+e\\ =Xb+Z\sum_{i=1}^{f}u_{i}+e \end{array}$$ (16)

$$\begin{array}{l} V=ZGZ^{\prime }+\sigma_{e}^{2}I_{N}=Z\left(\sum_{i=1}^{f}G_{i}\right)Z^{\prime }+\sigma_{e}^{2}I_{N}\\ =Z\left(\sum_{i=1}^{f}\sigma_{i}^{2}T_{i}T_{i}^{\prime }\right)Z^{\prime }+\sigma_{e}^{2}I_{N}=Z\left(\sum_{i=1}^{f}\sigma_{i}^{2}S_{i}\right)Z^{\prime }+\sigma_{e}^{2}I_{N} \end{array}$$ (17)

where **y** = N×1 column vector of phenotypic observations, **Z** = N×n incidence matrix allocating phenotypic observations to each individual = identity matrix for one observation per individual (N = n), N = number of observations, n = number of individuals, b=c×l column vector of fixed effects such as heard-year-season in dairy cattle, c = number of fixed effects, X=N×c model matrix, **b,e** = N×1 column vector of random residuals, $\sigma_{e}^{2}$ = residual variance, and $G=\sum_{i=1}^{f}G_{i}$ (Eq. 7). The phenotypic values (y) are assumed to follow a normal distribution with mean Xb and variance–covariance matrix of **V**. The methods described below for genomic estimation and prediction are based on the conditional expectation (CE) method, which is more efficient computationally than the methods based on mixed-model equations (MME) when the number of genetic effects is greater than the number of individuals (Da et al., 2014; Da, 2015).

For Model-I with 10 effect types, the genomic epistasis relationship matrices can be calculated using either EGERM or AGERM. However, EGERM or AGERM did not consider intra- and inter-chromosome genomic epistasis relationship matrices that are required by Model-II with 13 effect types. This research derives intra- and inter-chromosome genomic epistasis relationship matrices for both EGERM and AGERM.

### Intra- and inter-chromosome genomic epistasis relationship matrices

The main derivation of the intra- and inter-chromosome genomic epistasis relationship matrices is the partition of the numerator of a genomic epistasis relationship matrix into intra- and inter-chromosome numerators. The first step is to derive the intra-chromosome numerator, and the second step is to derive the inter-chromosome numerator as the difference between the whole-genome numerator and the intra-chromosome numerator. The last step is to divide the intra-chromosome numerator by the average of the diagonal elements of the intra-chromosome numerator and to divide the inter-chromosome numerator by the average of the diagonal elements of the inter-chromosome numerator. Using this procedure, intra- and inter-chromosome epistasis relationship matrices were derived for both EGERM and AGERM (Supplementary Text S1).

### Genomic best linear unbiased prediction and reliability

Based on the multifactorial genetic model of Eqs 16 and 17, the GBLUP of the genetic values of the $i^{th}$ effect type ( ${\hat{u}}_{i}$ ) and the best linear unbiased estimator (BLUE) or generalized least squares (GLS) estimator of fixed effect ( $\hat{b}$ ) are

$$\begin{array}{cc} {\hat{u}}_{i}=\sigma_{i}^{2}S_{i}Z^{\prime }V^{-1}\left(y-X\hat{b}\right)=\sigma_{i}^{2}S_{i}Z^{\prime }Py, & i=1,..., \end{array}f$$ (18)

$$\hat{b}={\left(X^{\prime }V^{-1}X\right)}^{-1}X^{\prime }V^{-1}y$$ (19)

where $P=V^{-1}-V^{-1}X{\left(X^{\prime }V^{-1}X\right)}^{-}X^{\prime }V^{-1}$ . The GBLUP of total genetic values of the n individuals is the summation of all types of genetic values:

$$\hat{g}=\sum_{i=1}^{f}{\hat{u}}_{i}.$$ (20)

Reliability of GBLUP is the squared correlation between the GBLUP of a type of genetic value and the unobservable true genetic value being predicted by the GBLUP. The expected accuracy of predicting the genetic values by the GBLUP is the square root of reliability or the correlation between the GBLUP of a type of genetic effect and the unobservable true genetic effects being predicted by the GBLUP. In the absence of validation studies for observed prediction accuracy, reliability or the expected prediction accuracy is the measure of prediction accuracy of the GBLUP. The reliability of the GBLUP of the total genetic value (Eq. 2) of the $j^{th}$ individual is

$$R_{gj}^{2}={\left[G\left(Z^{\prime }PZ\right)G\right]}^{jj}/G^{jj}$$ (21)

where $G=\sum_{i=1}^{f}G_{i}=\sum_{i=1}^{f}\sigma_{i}^{2}T_{i}T_{i}^{\prime }=\sum_{i=1}^{f}\sigma_{i}^{2}S_{i}$ (Eq. 7), $G^{jj}=\sum_{i=1}^{f}G_{i}^{jj}=\sum_{i=1}^{f}\sigma_{i}^{2}S_{i}^{jj}$ , and subscript or superscript jj denotes the $j^{th}$ diagonal element. The reliability formula for any or a combination of genetic values can be readily derived from Eq. 21, for example, the reliability of ${\hat{u}}_{3}$ (GBLUP of haplotype additive values) is obtained from Eq. 21 by deleting all terms except $G_{3}\left(Z^{\prime }PZ\right)G_{3}$ in the numerator and $\sigma_{3}^{2}S_{3}^{jj}$ in the denominator, with changes in the **V** and **P** matrices accordingly.

### Calculation of genomic best linear unbiased prediction and reliability for individuals with and without phenotypic observations separately

Two strategies are available for calculating GBLUP and the reliability of Eqs 20 and 21. Strategy-1 is a one-step strategy that includes all individuals with and without phenotypic observations in the same system of equations so that GBLUP and reliability are calculated simultaneously for all individuals. This strategy essentially augments the mixed model for individuals with phenotypic observations with a set of null equations consisting of “0”s but uses each genomic relationship matrix for all individuals, and these null equations and the use of the relationship matrix for all individuals do not affect the GBLUP, reliability, and heritability of individuals with phenotypic observations. The advantage of this one-step strategy is the simplicity of data preparation. For example, for a k-fold cross validation study, the phenotypic input file only needs to have k columns of the trait observations, with one column for each validation where the phenotypic observations for the validation individuals are set as “missing,” and the **X** and **Z** model matrices for the “missing” observations are set to zero. With this strategy, the genotypic data need to be processed only once. As the number of traits increases for validation studies, this one-step strategy becomes more appealing due to the savings in data preparation work. This strategy has been implemented in our computing tools of GVCBLUP (Wang et al., 2014), GVCHAP (Prakapenka et al., 2020), and EPIHAP (Liang et al., 2021, 2022). However, when the number of validation individuals or individuals without phenotypic values is large, each genomic relationship matrix ( $S_{i}$ matrix) is large, and the one-step strategy becomes more difficult as the number of individuals increases.

For large numbers of individuals without phenotypic observations, calculating GBLUP for individuals with and without phenotypic values separately is more efficient computationally than calculating GBLUP for all individuals in the same system of equations by applying Henderson’s BLUP for animals without phenotypic observations (Henderson, 1977) to GBLUP. Let $n_{1}$ = number of individuals with phenotypic observations, $n_{0}$ = number of individuals without phenotypic observations, $n=n_{1}+n_{0}$ , and let the $S_{i}$ matrix be partitioned as

$$\begin{array}{cc} S_{i}=\left[\begin{array}{cc} S_{i11} & S_{i10}\\ S_{i01} & S_{i00} \end{array}\right], & \;i=1,\ldots ,f \end{array}$$ (22)

where $S_{i11}=n_{1}\times n_{1}$ genomic relationship matrix of the genetic values of the $i^{th}$ effect type for individuals with phenotypic observations, $S_{i01}=n_{0}\times n_{1}=$ genomic relationship matrix of the genetic values of the $i^{th}$ effect type between individuals without phenotypic observations and individuals with phenotypic observations, $S_{i10}=S_{i01}^{\prime }=n_{1}\times n_{0}=$ genomic relationship matrix between individuals with phenotypic observations and individuals without phenotypic observations, and $S_{i00}$ = $n_{0}\times n_{0}$ genomic relationship matrix of the genetic values of the $i^{th}$ effect type for individuals without phenotypic observations. In Eqs 16 and 17, $y=y_{1}$ , the **Z** matrix needs to be changed to $Z=\left[\begin{array}{cc} Z_{1} & 0 \end{array}\right]$ , the $u_{i}$ vector partitioned as $u_{i}={\left[u_{i1}^{\prime },u_{i0}^{\prime }\right]}^{\prime }$ , and the **g** vector partitioned as $g=\left[g_{1}^{\prime },g_{0}^{\prime }\right]\prime$ , where $Z_{1}=N\times n_{1}$ incidence matrix allocating phenotypic observations to individuals with phenotypic observations, $0=N\times n_{0}$ incidence matrix with elements “0” connecting phenotypic observations to individuals without phenotypic observations. With these changes and Eq. 22, the **V** matrix of Eq. (17) can be re-written as

$$V=Z_{1}\left(\sum_{i=1}^{f}G_{i}\right)Z_{1}^{\prime }+\sigma_{e}^{2}I_{N}=Z_{1}\left(\sum_{i=1}^{f}\sigma_{i}^{2}S_{i11}\right)Z_{1}^{\prime }+\sigma_{e}^{2}I_{N}$$ (23)

and the GBLUP and reliability for individuals with and without phenotypic observations can be calculated as

$$\begin{array}{cc} {\hat{u}}_{i1}=\sigma_{i}^{2}S_{i11}Z_{1}^{\prime }V^{-1}\left(y_{1}-X\hat{b}\right)=\sigma_{i}^{2}S_{i11}Z_{1}^{\prime }Py_{1}, & i=1,...,f \end{array}$$ (24)

$${\hat{g}}_{1}=\sum_{i=1}^{f}{\hat{u}}_{i1}$$ (25)

$$R_{g1j}^{2}={\left[G_{11}\left(Z_{1}^{\prime }PZ_{1}^{\prime }\right)G_{11}\right]}^{jj}/G_{11}^{jj}$$ (26)

$$\begin{array}{cc} {\hat{u}}_{i0}=\sigma_{i}^{2}S_{i01}Z_{1}^{\prime }V^{-1}\left(y_{1}-X\hat{b}\right)=\sigma_{i}^{2}S_{i01}Z_{1}^{\prime }Py_{1}, & i=1,...,f \end{array}$$ (27)

$$\begin{array}{c} =\sigma_{i}^{2}S_{i01}S_{i11}^{-1}S_{i11}Z_{1}^{\prime }Py_{1}=G_{i01}G_{i11}^{-1}G_{i11}Z_{1}^{\prime }Py_{1}=G_{i01}G_{i11}^{-1}{\hat{u}}_{i1} \end{array}$$ (28)

$${\hat{g}}_{0}=\sum_{i=1}^{f}{\hat{u}}_{i0}$$ (29)

$$R_{g0j}^{2}={\left[G_{01}\left(Z_{1}^{\prime }PZ_{1}^{\prime }\right)G_{10}\right]}^{jj}/G_{00}^{jj}$$ (30)

where ${\hat{u}}_{i1}=n_{1}\times 1$ column vector of the GBLUP of the genetic values of the $i^{th}$ effect type for individuals with phenotypic observations, ${\hat{g}}_{1}=n_{1}\times 1$ column vector of the GBLUP of the total genetic values for individuals with phenotypic observations, $R_{g1j}^{2}=$ reliability for the $j^{th}$ individuals with phenotypic observations, ${\hat{u}}_{i0}=n_{0}\times 1$ column vector of the GBLUP of the genetic values of the $i^{th}$ effect type for individuals without phenotypic observations, ${\hat{g}}_{0}=n_{0}\times 1$ column vector of the GBLUP of the total genetic values for individuals without phenotypic observations, $R_{g0j}^{2}=$ reliability for the $j^{th}$ individuals without phenotypic observations, $G_{11}=\sum_{i=1}^{f}G_{i11}=\sum_{i=1}^{f}\sigma_{i}^{2}S_{i11}$ , $G_{01}=\sum_{i=1}^{f}G_{i01}=\sum_{i=1}^{f}\sigma_{i}^{2}S_{i01}$ , $G_{10}=\sum_{i=1}^{f}G_{i10}=\sum_{i=1}^{f}\sigma_{i}^{2}S_{i10}$ , $G_{11}^{jj}=\sum_{i=1}^{f}S_{i11}^{jj}\sigma_{i}^{2}$ , and $G_{00}^{jj}=\sum_{i=1}^{f}S_{i00}^{jj}\sigma_{i}^{2}$ .

Equations 27 and 28 yield identical results if $S_{i11}^{-1}$ exists. However, when the number of individuals is greater than the number of effect levels, such as the number of SNPs, $S_{i11}^{-1}$ in Eq. 28 does not exist, and Eq. 27 still can calculate the GBLUP. The usefulness of Eq. 28 is that it shows the GBLUP of individuals without phenotypic observations is the regression of the genetic values of individuals without phenotypic observations on the genetic values of individuals with phenotypic observations. The advantage of Eq. 27 is that it does not calculate $S_{i11}^{-1}$ and hence is unaffected by the singularity of $S_{i11}$ . Therefore, Eq. 27 is recommended for calculating GBLUP for individuals without phenotypic observation when the number of such individuals is large. The GBLUP calculations of Eqs 24, 27, and 28 do not involve the genomic relationship matrix among individuals without phenotypic observations $S_{i00}$ , which is much larger than $S_{i11}$ when $n_{1}$ is much larger than $n_{0}$ . The reliability calculation for individuals without phenotypic observations (Eq. 30) only uses the diagonal elements of $S_{i00}$ and not the entire $S_{i00}$ .

### Advantage of the integrated model over separate models

The multifactorial model of Eqs 16 and 17 integrating SNP, haplotype, and epistasis effects has the advantage of using more effect types and assessing each effect type based on the phenotypic values adjusted for all remaining effect types over separate models for SNP, haplotype, and epistasis effects that do not have a mechanism to adjust for effect types not in the model, and each uses a smaller number of genetic effects in the model.

This advantage of the multifactorial model assessing each effect type based on the phenotypic values adjusted for all remaining effect types can be shown using the MME version of the GBLUP for the $i^{th}$ effect type:

$$\begin{array}{l} {\hat{u}}_{i}={\left(Z_{i}^{\prime }Z_{i}+G_{i}^{-1}\right)}^{-1}\left[Z_{i}^{\prime }y-\left(Z_{i}^{\prime }X\hat{b}+\sum_{\begin{array}{l} j=1\\ j\neq i \end{array}}^{f}Z_{i}^{\prime }Z_{j}{\hat{u}}_{j}\right)\right]\\ ={\left(Z_{i}^{\prime }Z_{i}+G_{i}^{-1}\right)}^{-1}Z_{i}^{\prime }\left(y-X\hat{b}-\sum_{\begin{array}{l} j=1\\ j\neq i \end{array}}^{f}Z_{j}{\hat{u}}_{j}\right)={\left(Z_{i}^{\prime }Z_{i}+G_{i}^{-1}\right)}^{-1}Z_{i}^{\prime }y_{bu}^{*} \end{array}$$ (31)

$$\begin{array}{l} \hat{b}={\left(X^{\prime }X\right)}^{-}\left(X^{\prime }y-X^{\prime }\sum_{i=1}^{f}Z_{i}{\hat{u}}_{i}\right)\\ ={\left(X^{\prime }X\right)}^{-}X^{\prime }\left(y-\sum_{i=1}^{f}Z_{i}{\hat{u}}_{i}\right)={\left(X^{\prime }X\right)}^{-}X^{\prime }y_{u}^{*} \end{array}$$ (32)

where $y_{bu}^{*}=y-X\hat{b}-\sum_{\begin{array}{l} j=1\\ j\neq i \end{array}}^{f}Z_{j}{\hat{u}}_{j}$ = phenotypic observations adjusted for the fixed effects and all random genetic values except those of ${\hat{u}}_{i}$ , $y_{u}^{*}=y-\sum_{i=1}^{f}Z_{i}{\hat{u}}_{i}$ = phenotypic observations adjusted for all random genetic values, and ${\left(X^{\prime }X\right)}^{-}$ is a generalized inverse of $X^{\prime }X$ . Eq. 31 shows the MME version of ${\hat{u}}_{i}$ uses the phenotypic values adjusted for the GBLUP of all other effect types in the model. Since the MME version of ${\hat{u}}_{i}$ (Eq. 31) and $\hat{b}$ (Eq. 32) are identical to the CE version of ${\hat{u}}_{i}$ (Eq. 18) and $\hat{b}$ (Eq. 19), the CE version of ${\hat{u}}_{i}$ (Eq. 18) uses the phenotypic values adjusted for the GBLUP of all other effect types in the model even though the CE version does not do such adjustments explicitly.

### Genomic restricted maximum estimation (GREML) of variances and heritabilities

The estimation of variance components uses GREML and a combination of EM-REML and AI-REML algorithms of iterative solutions. EM-REML is slow but converges, whereas AI-REML is fast but fails for zero heritability estimates. In our GVCBLUP, GVCHAP, and EPIHAP computing packages that implement these two algorithms (Wang et al., 2014; Prakapenka et al., 2020; Liang et al., 2021), EM-REML is used automatically when AI-REML fails. The EM-REML iterative algorithm for the multifactorial model of Eqs 16 and 17 is

$$\begin{array}{cc} \sigma_{i}^{\mathrm{2(j+1)}}=\sigma_{i}^{\mathrm{2(j)}}yP^{\left(j\right)}ZS_{i}Z^{\prime }P^{\left(j\right)}y/tr\left(P^{\left(j\right)}ZS_{i}Z^{\prime }\right), & i=1,\ldots ,f \end{array}$$ (33)

$$\sigma_{e}^{\mathrm{2(j+1)}}=\sigma_{e}^{\mathrm{2(j)}}yP^{\left(k\right)}P^{\left(j\right)}y/tr\left(P^{\left(j\right)}\right)$$ (34)

where j = iteration number. The AI-REML iterative algorithm is an extension of the early formulations (Johnson and Thompson, 1995; Lee and van der Werf, 2006) to the multifactorial model of Eqs 16 and 17:

$$\theta^{\left(j+1\right)}=\theta^{\left(j\right)}+{\left(AI^{\left(j\right)}\right)}^{-1}\Delta^{\left(j\right)}$$ (35)

where $\theta ={\left(\sigma_{1}^{2}{,\sigma }_{2}^{2}{,...,\sigma }_{f}^{2},\sigma_{f+1}^{2}\right)}^{\prime }$ = (f+1)×1 column vector of variance–covariance components, $\sigma_{f+1}^{2}=\sigma_{e}^{2}$ = residual variance, $\Delta ={\left(\Delta_{1},\Delta_{2},...,\Delta_{f},\Delta_{f+1}\right)}^{\prime }$ = (f+1)×1 column vector of the partial derivatives of the log residual likelihood function with respect to each variance component, and j = iteration number. A typical term in Δ ( $\Delta_{i}$ ) and a typical term in **AI** ( ${AI}_{ik}$ ) are

$$\begin{array}{l} \Delta_{i}=-\frac{1}{2}tr\left(P\frac{\partial V}{\partial \sigma_{i}^{2}}\right)+\frac{1}{2}y^{\prime }P\frac{\partial V}{\partial \sigma_{i}^{2}}Py\\ =-\frac{1}{2}tr\left(PZS_{i}Z^{\prime }\right)+\frac{1}{2}y^{\prime }PZS_{i}Z^{\prime }Py,\;i=1,...,f+1 \end{array}$$ (36)

$$\begin{array}{l} {AI}_{ik}=\frac{1}{2}y^{\prime }P\frac{\partial V}{\partial \sigma_{i}^{2}}P\frac{\partial V}{\partial \sigma_{k}^{2}}Py\\ \;\begin{array}{cc} =\frac{1}{2}y^{\prime }PZ^{\prime }S_{i}Z^{\prime }PZS_{k}Z^{\prime }Py,\; & i,k=1,\ldots ,f+1 \end{array} \end{array}$$ (37)

where $S_{f+1}=I_{N}$ . For the full Model-I or Model-II, some effect types inevitably may have zero variances. In those cases, AI-REML (Equations 35–37) fails and EM-REML (Equations 33 and 34) still converges, although a slow convergence rate can be expected for the full Model-I or Model-II. Once the effect types with zero variances are removed from the model, AI-REML converges, and a fast convergence rate can be expected. The estimate of the genomic heritability for each type of genetic effects $\left(h_{i}^{2}\right)$ and the total heritability of all types of genetic effects $\left(H^{2}\right)$ are

$$\begin{array}{cc} h_{i}^{2}=\sigma_{i}^{2}/\sigma_{y}^{2} & \;i=1,\ldots ,f \end{array}$$ (38)

$$H^{2}=\sum_{i=1}^{f}h_{i}^{2}$$ (39)

where $\sigma_{y}^{2}=\sum_{i=1}^{f}\sigma_{i}^{2}{+\sigma }_{e}^{2}=$ phenotypic variance.

The heritability estimates of Eq. 38 can be used for model selection by removing effect types with heritability estimates below a user-determined threshold value from the prediction model. Since different traits may have different genetic architectures, we hypothesize that some traits may involve only a small number of the effect types, and some traits are more complex and involve more effect types; global epistasis may be more important than local high-order epistasis effects of haplotypes for some traits, whereas the reverse may be true for other traits, and some traits may be affected by both global high-order and local high-order epistasis effects. The heritability estimates from Eq. 37 provide an approach to evaluate these hypotheses and identify effect types relevant to the phenotypic variance, whereas the total heritability of Eq. 38 provides an estimate of the total genetic contribution to the phenotypic variance. In addition to the use of heritability estimates, prediction accuracy based on GBLUP can be used for model selection by requiring a threshold accuracy level for the effect type to be included in the prediction model, for example, we identified the A + A×A model to have the same accuracy of predicting the phenotypic values of daughter pregnancy rate as the full Model-I in U.S. Holstein cows (Liang et al., 2022).

### Estimation of pairwise epistasis effect and heritability

The heritability of an SNP, haplotype block, or pairwise epistasis effect is the contribution of the genetic effect to the phenotypic variance and is also the contribution to the heritability of the effect type, and is estimated through the GBLUP of the corresponding genetic effects. These heritability estimates can be used to identify genome locations with large contributions to the phenotypic variance. The estimation of pairwise epistasis effects and heritability is the most demanding computation because the pairwise epistasis model matrices must be creased and are no longer avoidable. Estimating the effects and heritabilities for third-order epistasis effects is computationally unfeasible and is not considered. GBLUPs of SNP, haplotype, and pairwise epistasis effects of Model-I (Supplementary Table S1) are calculated as

$$\begin{array}{cc} {\hat{\tau }}_{i}=\sigma_{i}^{2}T_{i}^{\prime }Z^{\prime }Py=T_{i}^{\prime }S_{i}^{-1}{\hat{u}}_{i} & \end{array}$$ (40)

where ${\hat{\tau }}_{i}$ is the m×1 column vector of SNP additive effects for i=1 , or SNP dominance effects for i=2 ; or b×1 column vector of haplotype additive effects for i=3 ; or ${(}_{2}^{m})\times 1$ column vector of A×A epistasis effects for i=4 , or $2{(}_{2}^{m})\times 1$ column vector of A×D epistasis effects for i=5 , or ${(}_{2}^{m})\times 1$ column vector of D×D epistasis effects for i=6 . For i=5 ; the order of A×D and D×A effects is determined by the order of the model matrices of those effects, that is, ${\hat{\tau }}_{5}={\left({\hat{\tau }}_{\alpha \delta }^{\prime },{\hat{\tau }}_{\delta \alpha }^{\prime }\right)}^{\prime }$ if $T_{5}=\left(T_{\alpha \delta },T_{\delta \alpha }\right)$ , or ${\hat{\tau }}_{5}={\left({\hat{\tau }}_{\delta \alpha }^{\prime },{\hat{\tau }}_{\alpha \delta }^{\prime }\right)}^{\prime }$ if $T_{5}=\left(T_{\delta \alpha },T_{\alpha \delta }\right)$ . The heritability of the $j^{th}$ effect of the $i^{th}$ effect type ( ${\hat{h}}_{ij}^{2}$ ) is estimated as a faction of the genomic heritability of the $i^{th}$ effect type ( ${\hat{h}}_{i}^{2}$ ):

$${\hat{h}}_{ij}^{2}=\left({\hat{\tau }}_{ij}^{2}/\sum_{i=1}^{m}{\hat{\tau }}_{ij}^{2}\right){\hat{h}}_{i}^{2}=\left({\hat{\tau }}_{ij}^{2}/{\hat{\tau }}_{i}^{\prime }{\hat{\tau }}_{i}\right){\hat{h}}_{i}^{2}={\hat{\sigma }}_{ij}^{2}/{\hat{\sigma }}_{y}^{2}$$ (41)

where ${\hat{\tau }}_{ij}=$ the $j^{th}$ effect of ${\hat{\tau }}_{i},$ ${\hat{\sigma }}_{i}^{2}=$ estimated variance of the $i^{th}$ effect type, ${\hat{\sigma }}_{ij}^{2}=$ estimated variance of the $j^{th}$ effect of the $i^{th}$ effect type, and ${\hat{h}}_{i}^{2}$ = genomic heritability of the $i^{th}$ effect type defined by Equation (52). For proving Equation 57, ${\hat{\sigma }}_{i}^{2}$ and ${\hat{\sigma }}_{ij}^{2}$ can be formulated based on the method of mixed-model equations (MME):

$${\hat{\sigma }}_{i}^{2}={\hat{\tau }}_{i}^{\prime }{\hat{\tau }}_{i}/\left[m_{i}-,{tr(C}^{ii}{)\lambda }_{i}\right]=\sum_{j=1}^{m_{i}}\tau_{ij}^{2}/\left[m_{i}-{tr(C}^{ii}{)\lambda }_{i}\right]=\sum_{j=1}^{m_{i}}{\hat{\sigma }}_{ij}^{2}$$ (42)

$${{\hat{\sigma }}_{ij}^{2}=\hat{\tau }}_{ij}^{2}/\left[m_{i}-{tr(C}^{ii}{)\lambda }_{i}\right],$$ (43)

where $C^{ii}$ is the submatrix in the inverse or generalized inverse of the coefficient matrix of the MME corresponding to the $i^{th}$ effect type, $m_{i}$ = number of effects of the $i^{th}$ effect type, and $\lambda_{i}={\hat{\sigma }}_{e}^{2}/{\hat{\sigma }}_{i}^{2}$ . Dividing Eq. 43 by ${\hat{\sigma }}_{y}^{2}$ and multiplying by ${\hat{\sigma }}_{i}^{2}/{\hat{\sigma }}_{i}^{2}$ yield Eq. 41:

$${\hat{h}}_{ij}^{2}=\left({\hat{\sigma }}_{ij}^{2}/{\hat{\sigma }}_{i}^{2}\right)\left({\hat{\sigma }}_{i}^{2}/{\hat{\sigma }}_{y}^{2}\right)=\left({\hat{\sigma }}_{ij}^{2}/{\hat{\sigma }}_{i}^{2}\right)\left({\hat{\sigma }}_{i}^{2}/{\hat{\sigma }}_{y}^{2}\right)=\left({\hat{\tau }}_{ij}^{2}/\sum_{i=1}^{m}{\hat{\tau }}_{ij}^{2}\right){\hat{h}}_{i}^{2}=\left({\hat{\tau }}_{ij}^{2}/{\hat{\tau }}_{i}^{\prime }{\hat{\tau }}_{i}\right){\hat{h}}_{i}^{2}=\left({\hat{\sigma }}_{ij}^{2}/{\hat{\sigma }}_{y}^{2}\right).$$

It is readily seen that the sum of all heritability estimates of the $i^{th}$ effect type is the genomic heritability of the $i^{th}$ effect type: $\sum_{i=1}^{m_{i}}{\hat{h}}_{ij}^{2}={\hat{h}}_{i}^{2}$ . It is of note that Eqs 42 and 43 using MME are only for proving Eq. 41. The MME method is computationally prohibitive for estimating genetic effects and their variances under the multifactorial model, although the MME method yields results identical to the CE method, which is computationally feasible for genomic estimation and prediction under the multifactorial model.

### Comparison between exact and approximate genomic epistasis relationship matrices

We evaluated the differences between AGERM and EGERM in genomic heritability estimates and prediction accuracies using a publicly available swine genomics data set that had 3,534 animals from a single PIC nucleus pig line with five anonymous traits and 52,842 genotyped and imputed autosome SNPs after filtering by requiring minor allele frequency (MAF) > 0.001 and proportion of missing SNP genotypes < 0.100 (Cleveland et al., 2012). The EGERM followed the method used by Jiang and Reif (2020), and the AGERM methods are described in Supplementary Text S1. The heritability results showed that EGERM had slightly higher heritability estimates than AGERM except for the A×A heritability of T3, where AGERM had a slightly higher estimate than EGERM (0.280 vs. 0.278, Table 1). From Table 1, effect type with nonzero heritability estimates was included in the prediction model for evaluating the observed prediction accuracy as the correlation between the GBLUP of genotypic values and the phenotypic values in each validation population and then averaged over all 10 validation populations. The results showed that AGERM and EGERM had the same prediction accuracy for this swine sample (Table 2). A disadvantage of EGERM is the computing time for the construction of EGERM, about 9.51 times as much time for pairwise relationship matrices, 8.29 as much time for third-order and 9.44 times as much time for fourth-order as required for AGERM (Table 3). However, computing time is not the deciding factor for choosing between the exact and approximate methods because the multi-node approach that calculates each genomic relationship matrix in pieces and adds those pieces together can reduce the computing time to an acceptable level when multiple threads/cores are available, and the two-step strategy can be used so that each genomic relationship is calculated only once for different traits and validation populations (Prakapenka et al., 2020). Prediction accuracy is the ultimate deciding factor in choosing between different methods. We reported results of comparing AGERM and EGERM using 60,671 SNPs and 22,022 first-lactation Holstein cows with phenotypic observations of daughter pregnancy rates, showing that AGERM and EGERM had the same heritability estimates and prediction accuracy, but EGERM required 21 times as much computing time as that required by AGERM, which required 1.32 times as much time for the genomic additive relationship matrix (Liang et al., 2022). The combined results of the swine and Holstein samples indicated that EGERM and AGERM had similar results and that the computing difficulty of EGERM over AGERM increased rapidly as the sample size increased. Given the computing difficulty of EGERM and the negligible differences between EGERM and AGERM in prediction accuracy, AGERM should be favored for its mathematical simplicity and computing efficiency, at least for samples with 50,000 SNPs or more.

**TABLE 1:** Genomic heritability estimates of additive, dominance, and epistasis effects up to the third-order for five traits in a swine population.

	Trait				
	T1	T2	T3	T4	T5
Effect	Exact genomic epistasis relationship matrices (EGERM)				
A	0.023	0.217	0.131	0.336	0.366
D	0.000	0.013	0.000	0.000	0.052
A×A	0.046	0.186	0.278	0.017	0.054
A×D	0.000	0.000	0.091	0.000	0.000
D×D	0.000	0.000	0.091	0.000	0.000
A×A×A	0.000	0.000	0.000	0.000	0.000
A×A×D	0.000	0.000	0.079	0.000	0.000
A×D×D	0.000	0.000	0.102	0.000	0.000
D×D×D	0.000	0.000	0.117	0.000	0.000
Total heritability	0.069	0.416	0.889	0.354	0.471
Effect	Approximate genomic epistasis relationship matrices (AGERM)				
A	0.022	0.215	0.139	0.329	0.360
D	0.000	0.013	0.000	0.000	0.051
A×A	0.043	0.176	*0.280*	0.016	0.050
A×D	0.000	0.000	0.091	0.000	0.000
D×D	0.000	0.000	0.090	0.000	0.000
A×A×A	0.000	0.000	0.000	0.000	0.000
A×A×D	0.000	0.000	0.075	0.000	0.000
A×D×D	0.000	0.000	0.095	0.000	0.000
D×D×D	0.000	0.000	0.109	0.000	0.000
Total heritability	0.065	0.404	0.879	0.346	0.461

**TABLE 2:** Observed prediction accuracy of epistasis models relative to the additive model for five traits in a swine population.

	Trait				
	T1	T2	T3	T4	T5
Prediction accuracy of SNP model					
A	0.066	0.495	0.326	0.468	0.493
A + D	0.056	0.495	0.326	0.468	0.496
Epistasis model	A + AA	A + D + AA	A + AA + AD + DD+		
AAD + ADD + DDD	A + AA	A + D + AA			
EGERM					
Prediction accuracy	0.063	0.498	0.336	0.468	0.497
Accuracy increase (%)	−4.545	0.606	3.067	0.000	0.202
AGERM					
Prediction accuracy	0.063	0.498	0.336	0.468	0.497
Accuracy increase (%)	−4.545	0.606	3.067	0.000	0.202

“Prediction accuracy” is the observed prediction accuracy calculated as the correlation between the GBLUP of genotypic values and the phenotypic values in each validation population and then averaged over all 10 validation populations. “Accuracy increase” is the percentage increase of the observed prediction accuracy of the epistasis model over the observed prediction accuracy of the best SNP model, which was the additive model (A) for T1–T4 and the A + D model for T5. A = additive effects, D = dominance effects, AA = A×A effects, AD = A×D effects, DD = D×D effects, AAA = A×A×A effects, AAD = A×A×D effects, ADD = A×D×D dominance effects, and DDD = D×D×D dominance effects.

**TABLE 3:** Computing time (in seconds) for the construction of exact and approximate genomic epistasis relationship matrices for a swine population with 3,534 pigs and 52,843 SNPs using 20 threads of the Mangi supercomputer of the Minnesota Supercomputer Institute at the University of Minnesota.

Genomic epistasis relationship matrices	Pairwise	Third-order	Fourth-order
EGERM	666	796	1,256
AGERM	70	96	133
EGERM/AGERM	9.51	8.29	9.44

### Numerical demonstration

The methods of genomic epistasis relationship matrices based on the additive and dominance model matrices, GREML, GBLUP and reliability, and estimation of effect heritability are demonstrated using an R program (DEMO.R) and a small artificial sample for the convenience of reading the numerical results (Supplementary Text S2 and R program). Because of the artificial nature and the extremely small sample size, this numerical demonstration does not have any genetic and methodology implications and is for showing calculations of the methods only. This R program is an extension of the R demo program of GVCHAP that integrates SNP and haplotype effects and has a computing pipeline for producing the input haplotype data from the SNP data (Prakapenka et al., 2020).

## Conclusion

The multifactorial methods with SNP, haplotype, and epistasis effects up to the third-order provide an approach to investigate the contributions of global low-order and local high-order epistasis effects to the phenotypic variance and the accuracy of genomic prediction. Genomic heritability of each effect type from GREML and prediction accuracy from validation studies using GBLUP can be used jointly to identify effect types contributing to the phenotypic variance and the accuracy of genomic prediction, and the GBLUP for the multifactorial model with selected effect type can be used for genomic evaluation. With many capabilities, including the use of intra- and inter-chromosome separately, the multifactorial methods offer a significant methodology capability to investigate and utilize complex genetic mechanisms for genomic prediction and for understanding the complex genome–phenome relationships.

## Data Availability

Publicly available datasets were analyzed in this study. These data can be found at: https://academic.oup.com/g3journal/article/2/4/429/6026060#supplementary-data.
